# Phenotype-driven strategies for exome prioritization of human Mendelian disease genes

**DOI:** 10.1186/s13073-015-0199-2

**Published:** 2015-07-30

**Authors:** Damian Smedley, Peter N. Robinson

**Affiliations:** Skarnes Faculty Group, Wellcome Trust Sanger Institute, Hinxton, UK; Institute for Medical Genetics and Human Genetics, Charité-Universitätsmedizin Berlin, Berlin, Germany; Max Planck Institute for Molecular Genetics, Ihnestrasse, 14195 Berlin, Germany; Berlin Brandenburg Center for Regenerative Therapies (BCRT), Charité-Universitätsmedizin Berlin, Augustenburger Platz, 13353 Berlin, Germany; Institute for Bioinformatics, Department of Mathematics and Computer Science, Freie Universität Berlin, Takustrasse, 14195 Berlin, Germany

## Abstract

Whole exome sequencing has altered the way in which rare diseases are diagnosed and disease genes identified. Hundreds of novel disease-associated genes have been characterized by whole exome sequencing in the past five years, yet the identification of disease-causing mutations is often challenging because of the large number of rare variants that are being revealed. Gene prioritization aims to rank the most probable candidate genes towards the top of a list of potentially pathogenic variants. A promising new approach involves the computational comparison of the phenotypic abnormalities of the individual being investigated with those previously associated with human diseases or genetically modified model organisms. In this review, we compare and contrast the strengths and weaknesses of current phenotype-driven computational algorithms, including Phevor, Phen-Gen, eXtasy and two algorithms developed by our groups called PhenIX and Exomiser. Computational phenotype analysis can substantially improve the performance of exome analysis pipelines.

**Electronic supplementary material:**

The online version of this article (doi:10.1186/s13073-015-0199-2) contains supplementary material, which is available to authorized users.

## Disease-associated gene discovery and genomic diagnostics

It seems fair to say that next-generation sequencing (NGS)-based diagnostics are revolutionizing the way that rare diseases are diagnosed and researched. For example, programs such as Care4Rare [1], the program at the Centers for Mendelian Genomics [2], and the Undiagnosed Diseases Program of the National Institutes for Health [3] have developed computational and clinical frameworks for the efficient identification of novel genes implicated in disease. Furthermore, clinical groups have shown the utility of exome and genome sequencing in improving the diagnosis of rare genetic diseases [4–11]. The UK 100,000 Genomes Project, which aims to transform the way that genomics is used in the National Health Service (NHS), is focused on the areas of rare disease, infectious disease and cancer. This project has recently reported the first successful diagnoses of patients using exome sequencing [12] (Box 1). Detailed clinical phenotyping is a keystone of the UK 100,000 Genomes Project's strategy; the aim is to use phenotypic analysis to guide the interpretation of genome sequence data that cover at least 95 % of the genome at 15-fold or better.

Many clinical centers are now using whole exome sequencing (WES). This process relies on oligonucleotide probes to capture (hybridize to) the target exonic sequences from fragmented total genomic DNA, followed by enrichment and NGS of the targeted sequences [13]. WES is typically performed using kits that aim to capture all exonic and flanking sequences and may also include probes to target microRNA and other sequences of interest [14]. Recent large-scale clinical WES studies have reported a successful molecular diagnosis in up to 25 % of cases in large cohorts of unselected, consecutive patients [6–8, 15]. Despite this progress, it remains difficult to identify causative mutations in the genomes of many patients.

A number of strategies have emerged to rank the variants and the genes that they affect, with those most likely to cause disease ranked highest, through a process termed gene prioritization [16–18]. Current approaches towards gene prioritization include simultaneously sequencing multiple affected individuals and searching for genes that are affected in all or most individuals [17], linkage analysis [19], and various forms of network analysis [20]. The first two strategies identify specific genes or genomic intervals as candidates, whereas network approaches generate a relative likelihood that every gene in the genome is causal. An additional strategy that is proving particularly successful uses knowledge of the patient’s phenotype to assess candidate sequences.

In this review, we provide an overview of the current tools that use computational analysis of the phenotype as a major component of their exome prioritization procedures. We explain how phenotype-driven analysis of exome data can be used to filter out common variants and those deemed to be non-pathogenic. We also present a number of recently published tools that substantially improve the analysis of WES data by incorporating phenotypic features into their prioritization procedures, and compare their strengths and weaknesses.

## Variant annotation and filtering

Exome analysis of the tens of thousands of sequence variants typically found in any individual usually begins with filtering out of target and high-frequency variants. In many cases, the remaining variants are filtered or prioritized on the basis of their predicted pathogenicity. An essential step in the interpretation of these data is the annotation of these variants with respect to their potential effects on genes and transcripts; this requires the translation of variant-describing semantics in the Variant Call Format (VCF), which reflects the chromosomal coordinates of each variant (for example, chr10:g.123256215T>G), into gene-based variant annotations (such as c.518A>C; p.Glu173Ala in the gene *FGFR2*). This is necessary because evaluation of a variant in a diagnostic context almost always requires assessment of the potential effects of variants on gene products [21].

Several annotation tools offer additional functionality that allows variants to be filtered according to their population frequency and variant class. For instance, ANNOVAR [22] annotates variants relative to a number of popular gene sets to identify the functional consequence of the mutation; for example, new amino acid (missense) or stop-codon (nonsense) mutations can result from a non-synonymous point mutation. In addition, this tool can filter variants to produce a more manageable set of candidates on the basis of various criteria, such as excluding any common single nucleotide polymorphisms (SNPs) present in dbSNP or present with a minor allele frequency (MAF) more than 1 % in the 1000 Genomes Project [23] or NHLBI-ESP 6500 exome project (ESP) datasets. Other sources of data that can be used for prioritization include deleteriousness scores precomputed using the variant-analysis tools Sorting Intolerant from Tolerant (SIFT) [24], Polymorphism Phenotyping (PolyPhen) [25], Genomic Evolutionary Rate Profiling (GERP) [26], and Combined Annotation-Dependent Depletion (CADD) [27] (Box 2). Finally, the exome annotation tool Jannovar can implement the expected inheritance model for further filtering [21]. The Variant Effect Predictor [28] of the European Bioinformatics Institute (EBI) can be used through either an online interface, a downloadable Perl command-line tool or a scalable web service such as RESTful. Variants can be input in a number of formats (VCF, Human Genome Variation Society (HGVS) and so on) and the functional consequence annotated using a number of transcript sets (Ensembl, Gencode or Refseq). Filters can be set to exclude non-coding variants or common variants above a certain MAF in the variant populations provided by the1000 Genomes Project [23], the Exome Sequencing Project [29], or the Exome Aggregation Consortium [30]. The output also includes predicted deleteriousness scores from SIFT and PolyPhen.

## Phenotype-based exome analysis tools

When the diagnosis is not known in advance, or if a novel disease gene is being sought, computational phenotype analysis can serve to assess each candidate gene’s relevance to the clinical abnormalities observed in the patient(s). Although other ontologies or terminologies that represent phenotypes exist (such as SNOMED CT, MeDRA, London Dysmorphology Database, POSSUM, PhenoDB, ICD-9/10/11) [31] the current applications in this field make use of the Human Phenotype Ontology (HPO) database, which aims to provide a computable representation of the clinical abnormalities observed in human disease [32]. A number of algorithms have been developed to estimate the similarity between two diseases based on their phenotypic features encoded using HPO terms [33]. These algorithms can be adapted to measure the similarity between a set of query terms representing the clinical manifestations observed in a patient and those representing each of the diseases in a database [34–37]. The algorithms below utilize an assessment of clinical similarity to prioritize candidate genes.

### eXtasy

eXtasy [38] takes a data integration approach (genomic data fusion [39]) to variant prioritization. To generate an overall prediction of causality, ten different measures of variant deleteriousness that are available from existing tools and databases, along with a gene haploinsufficiency prediction score, are combined with a phenotype-specific gene score. The phenotype-based method takes all disease genes known to be associated with a particular HPO term or terms from Phenomizer [37] and scores the similarity of each candidate gene in the exome to this gene set using the Endeavour algorithm [39]. Endeavour uses various measures of gene similarity, such as sequence similarity and co-expression, as well as involvement in the same protein–protein interactions or pathways. A Random Forest algorithm is used to produce a single combined candidacy score from all of these sources of evidence. For variants that are missing data from any of the methods, an imputed score is calculated that ignores haploinsufficiency and uses median values across all variants for the missing deleteriousness scores.

Receiver operating characteristic (ROC) analysis was used to assess the ability of eXtasy to discriminate disease-causing from rare control variants or common polymorphisms. This analysis showed substantial improvement when compared with classical deleterious prediction methods such as PolyPhen, SIFT, MutationTaster and CAROL. Currently, eXtasy only performs prioritization of non-synonymous variants but when public datasets that are sufficiently large for training become available, it will be expanded to include mitochondrial, noncoding, synonymous and nonsense variants, as well as mutations around the splice junction that affect splicing and insertion and deletion of base mutations (indels). eXtasy performs no filtering, so it is recommended that the exome is pre-filtered to remove off-target or common (MAF > 1 %) variants. eXtasy is available for online use or download [40].

### Phevor: Phenotype Driven Variant Ontological Re-ranking tool

Phevor [41] takes the outputs of variant-prioritization tools such as ANNOVAR or the Variant Annotation, Analysis, Search Tool (VAAST) [42] and then prioritizes the remaining genes using phenotype, gene function and disease data. This knowledge comes from publically available gene annotation sets using various biomedical ontologies such as the HPO, Mammalian Phenotype Ontology (MPO) [43, 44], Disease Ontology (DO) [45], and Gene Ontology (GO) [46]. Users specify a list of terms from one or more of HPO, DO, MPO, GO or Online Inheritance in Man (OMIM) [47] that characterize what is known about the patient. Phevor then generates a list from genes that have been annotated with these terms or their parent terms if no gene annotations exist. Next, it identifies terms in the other ontologies that are annotated to these genes and the process is repeated to expand the gene list. Thus, concepts in different ontologies are related through their annotation of the same gene. Finally, each gene receives a score based on propagation from the seed nodes in each ontology and a combination procedure across the scores from the various ontologies. The final Phevor score combines the ranking information for the variant prioritization tool (or *P*-value from VAAST) with this gene score.

Benchmarking of Phevor on simulated disease exomes, based on in-house generated exomes, demonstrated a considerable improvement over variant prioritization methods such as ANNOVAR and VAAST, with 95–100 % of the exomes having the causative variant in the top ten candidates. Three case studies where Phevor was used to identify disease-causing alleles have also been presented. Phevor is available for online use only [48].

### Phen-Gen

Phen-Gen [49] uses a Bayesian framework to compare predicted deleterious variants in the patient’s exome and known patient symptoms to prior knowledge of human disease-gene associations and gene interactions. Coding variants are analyzed using a unifying framework to predict the damaging impact of non-synonymous, splice-site and indel variants. Phen-Gen also allows a genome-wide approach in which evolutionary conservation and Encyclopedia of DNA Elements (ENCODE)-predicted functionality and proximity to coding sequences are used to score non-coding variants.

Any variant that has a MAF above 1 % is removed from further analysis. Healthy individuals contain many damaging mutations and the fact that this ability to tolerate mutations varies from gene to gene is also taken into account using a null model. This model uses the observed variants from the 1000 Genomes Project to generate a null distribution under either a dominant or recessive inheritance model for each gene. Genes are only retained for further analysis if the predicted damaging score for the variants exceeds that seen for 99 % of the 1000 Genomes dataset.

These remaining genes are then analyzed using the Phenomizer algorithm to match semantically the patient’s phenotypes encoded using HPO to known disease-gene associations. The role of novel (non-disease genes) is assessed by identifying functionally related genes using a random-walk-with-restart algorithm over a gene interaction network. Phenotype matches are distributed to these novel genes across the network such that the disease gene hub gets the majority (90 %) of the score and other genes get a share of the remainder, according to their proximity to the disease gene.

Benchmarking using simulated exomes that were based on 1000 Genomes Project data showed that the correct disease variant was obtained as the top hit in 88 % of samples. Using a strategy in which known associations were masked to simulate the discovery of novel associations, performance figures of 56 % and 89 % were obtained for dominant and recessive disorders, respectively. In an evaluation using real patient data, 11 trios with recessive or X-linked intellectual disability were analyzed and 81 % of the reported genes were in the top ten candidates. Phen-Gen is available for online use or download [49].

### Exomiser

The original implementation of Exomiser [50] used semantic similarity comparisons between patient phenotypes and mouse phenotype data for each candidate gene in the exome. The PhenoDigm [51] algorithm is used to score each gene from 0 to 1, where 1 represents the perfect match and genes with no data received a default score of 0.6. This phenotype score is combined with a variant score that is based on the allele rarity in the 1000 Genomes Project and ESP datasets together with predictions of deleteriousness from PolyPhen, SIFT and MutationTaster.

Benchmarking on simulated exomes based on 1000 Genomes Project data showed that 66 % of cases had the causative variant as the top hit under a dominant model and 83 % under a recessive model [50].

Exomiser has been improved subsequently to include comparison with human and fish phenotypes, as well as use of a random-walk with restart to score genes with no phenotype data (genes are scored based on proximity in the StringDB interaction network to other genes that do show phenotypic similarity to the patient data) [20]. Exomiser is available as an online web service [52] or for download as a command-line tool. Installation simply involves unzipping the download.

### PhenIX

PhenIX [5] uses the same software framework as Exomiser but instead of using human, mouse, fish, and protein–protein association data, this tool is restricted to comparisons between patient phenotypes and known disease gene phenotypes. This simplification is made because PhenIX is intended for diagnostic tasks when only known disease genes can be reported. In addition, the semantic similarity algorithm uses the Phenomizer algorithm [37].

Benchmarking on sequence files generated from a target enrichment panel that was based on known disease-associated genes revealed that 97 % of samples had the inserted variant as the top hit, regardless of inheritance model. The same performance was observed when using 1000 Genomes Project exomes.

PhenIX is available in the same downloadable library as Exomiser and has the same filtering options. In addition it can be used from its website [52].

## Comparison of exome prioritization tools

Table 1 summarizes the main features of the software solutions described above. For clinicians and many researchers, a well-designed web interface solution is best in terms of usability. Installation of the command-line versions of the tools will be difficult or off-putting for many such users. Nevertheless, web-based solutions present security issues in that patient exomes have to be uploaded onto external servers. To counter this, publically available, secure, cloud-based versions or easy-to-install local clients would be welcomed in the future. By contrast, for many medium-to-large projects, the primary users of these tools are going to be the bioinformatics teams that support clinical researchers. For these users, a command-line version that can be integrated into their pipelines is the most useful platform; for example, some of the tools can take as input VCF files from one program and can output VCF that can feed into another.

**Table 1:** Comparison of exome analysis tools

Software	Exome input	Types of variant analyzed	Availability	Software approach
VEP	Various including VCF, pileup, HGVS notations	All	Website, command line and REST service	Filtering by allele frequency and deleteriousness scores (SIFT, PolyPhen)
ANNOVAR	Various including multi-sample VCF	All	Command line	Filtering by allele frequency, inheritance model and deleteriousness scores (SIFT, PolyPhen, MutationTaster, MutationAssessor, LRT, FATHMM, MetaSVM, MetaLR, GERP++, PhyloP, SiPhy, CADD)
eXtasy	Single sample VCF	Non-synonymous only	Website and command line	Prioritization based on a Random Forest score from combined deleteriousness scores (CAROL, LRT, MutationTaster, PhastCons, PhyloP, PolyPhen, SIFT), haploinsufficiency, and similarity of the gene to genes annotated with the input Human Phenotype Ontology (HPO) phenotypes as measured by sequence similarity, co-expression, and involvement in the same pathway or protein–protein interactions
Phevor	Pre-filtered VAAST or ANNOVAR files or functionally annotated multi-sample VCF	All	Website	Prioritization based on semantic similarity of each candidate gene to genes annotated with the input set of ontology terms taken from HPO, Mammalian Phenotype Ontology (MPO), Disease Ontology (DO), and Gene Ontology (GO)
Phen-Gen	Multi-sample family VCF	All	Website and command line	Filtering by inheritance model and stringency or reentrance. Prioritization based on predicted variant impact and semantic phenotypic similarity between HPO input and HPO-annotated diseases associated with each exomic candidate or its neighbors in an interaction network
PhenIX	Multi-sample family VCF	All coding	Website and command line	Filtering by allele frequency, variant quality, and inheritance model. Prioritization based on predicted deleteriousness (SIFT, PolyPhen, MutationTaster), allele frequency and semantic phenotypic similarity between HPO input and HPO-annotated diseases associated with each exomic candidate
Exomiser	Multi-sample family VCF	All coding	Website and command line	Filtering by allele frequency, variant quality, deleteriousness scores and inheritance model. Prioritization based on predicted deleteriousness (SIFT, PolyPhen, MutationTaster), allele frequency and semantic phenotypic similarity between HPO input and HPO-annotated diseases, MPO-annotated mouse and Zebrafish Phenotype Ontology (ZPO)-annotated fish models associated with each exomic candidate or its neighbors in an interaction network

Abbreviations: *CADD* Combined Annotation-Dependent Depletion, *GERP* Genomic Evolutionary Rate Profiling, *HGVS* Human Genome Variation Society, *HPO* Human Phenotype Ontology, *LRT* likelihood ratio test (LRT), *PolyPhen* Polymorphism Phenotyping, *REST* Representational State Transfer, *SIFT* Sorting Intolerant from Tolerant, *VAAST* Variant Annotation, Analysis, Search Tool, *VCF* variant call format

**Fig. 1:**
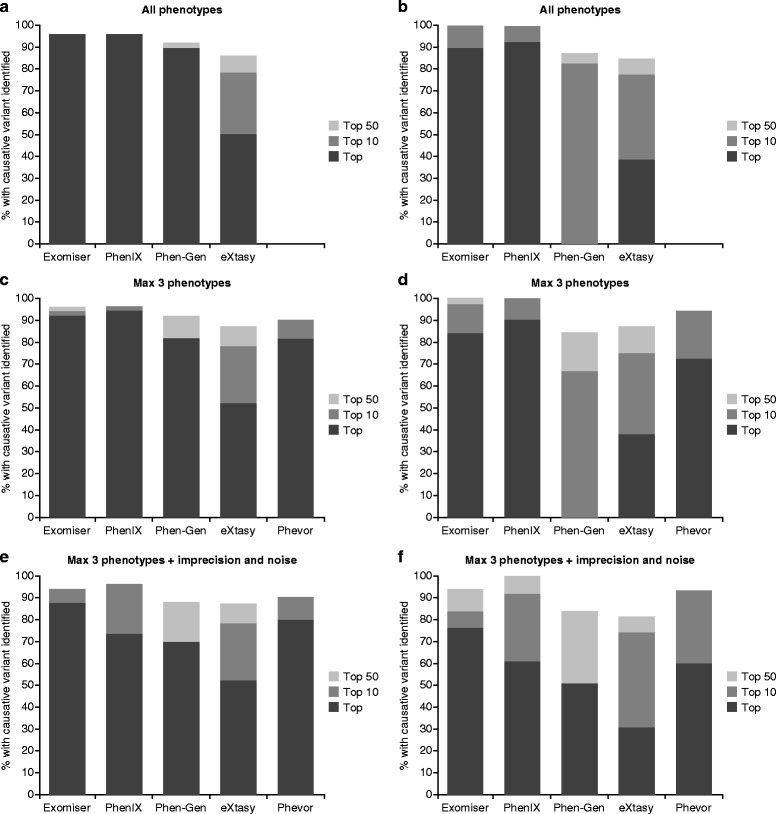
Benchmarking of all phenotype-based exome analysis tools on 1000 Genomes Project or in-house exomes. Exomes were generated by randomly inserting known disease variants from the Human Genome Mutation Database (HGMD) into either (**a**, **c**, **e**) 50 unaffected exomes from the 1000 Genomes Project or (**b**, **d**, **f**) 50 in-house generated exomes. These exomes were analyzed using each tool and the ability of each tool to rank the causative variant as the top hit, in the top 10 or top 50 was recorded. Default settings, along with filtering with a minor allele frequency cutoff of 1 %, were used for all tools. Analysis was performed using (**a**, **b**) all phenotype annotations (**c**, **d**) just three of the terms chosen randomly, or (**e**, **f**) with two of these three terms made less-specific and two random terms from the whole of the Human Phenotype Ontology (HPO) added

To further compare these tools, benchmarking was performed on 50 simulated disease exomes, generated by randomly adding known non-synonymous disease variants (two copies for recessive diseases and one for dominant) from the Human Genome Mutation Database (HGMD) to either 50 randomly chosen unaffected exomes from the 1000 Genomes Project or 50 exomes generated by us in-house (Fig. 1). The diseases and variants used for the benchmarking of the 50 exomes in Fig. 1 are detailed in Additional file 1. Two background sources of exome data were used because the 1000 Genomes Project exomes can over-predict the performance that will be obtained for real patient exomes. This is because many of the tools utilize the allele frequency data from the 1000 Genomes Project for filtering and prioritization. Data from the 1000 Genomes Project variants have also been used to train some of the algorithms. In addition, real patient exomes typically contain many more variants than the conservatively called 1000 Genomes Project exomes; for example, our in-house generated exomes contain 140,000–231,000 variants compared to 24,000–42,000 in the 1000 Genomes Project exomes.

Exomiser and PhenIX were run from the command-line with the default settings and MAF filter set to <1 % and the appropriate inheritance model specified. Phen-Gen was run from the command line, again with the inheritance model specified. EXtasy was run from the command line using just the phenotypes as additional arguments. EXtasy does not perform any variant filtering, so to allow a better comparison with the other tools, we ran it on the filtered variants from Exomiser. Phevor is also just a variant prioritizer and relies on a filtered exome from software such as VAAST or ANNOVAR. Hence, we used the output of ANNOVAR's variant_reduction.pl script with the default settings along with specification of the inheritance model. Table 2 shows the average gene counts before and after filtering by these various strategies.

**Table 2:** Number of genes per benchmarked sample

	1000 Genomes Project exomes		In-house exomes	
	AD	AR	AD	AR
Before filtering	10542 ± 783	10631 ± 802	19235 ± 916	19712 ± 976
Exomiser filtered	388 ± 110	38 ± 11	973 ± 104	557 ± 74
PhenIX filtered	388 ± 110	38 ± 11	973 ± 104	557 ± 74
Exomiser filtered for eXtasy analysis	388 ± 110	38 ± 11	973 ± 104	557 ± 74
Phen-Gen filtered	100 ± 34	5 ± 4	665 ± −86	331 ± 70
Annovar filtered for Phevor analysis	88 ± 36	2 ± 1	372 ± 61	52 ± 17

Abbreviations: *AD* autosomal dominant, *AR* autosomal recessive

HPO annotations for the disease under consideration were included in the prioritization analysis for each software. We assessed performance when using: (a) all available phenotypes, (b) a maximum of three phenotypes randomly chosen from the annotations, (c) the same three phenotypes but with two promoted to the less-specific parent term and two false-positive terms randomly chosen from the whole of HPO. Phevor only allows up to five HPO terms, so only the latter two options were tested for this tool.

**Fig. 2:**
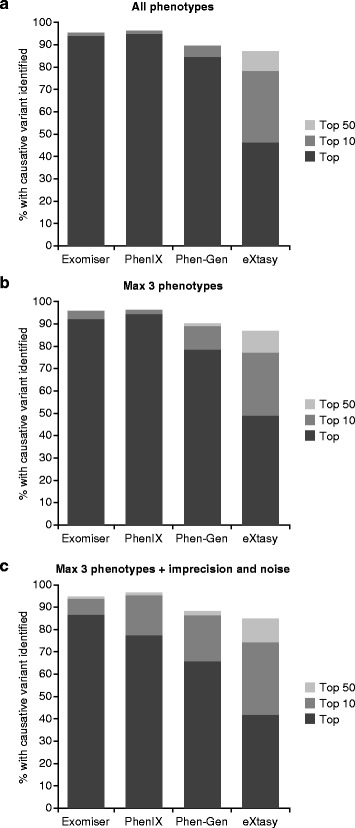
Benchmarking of command-line exome analysis software. Exomes were generated by randomly inserting known disease variants from the Human Genome Mutation Database (HGMD) into 1000 unaffected exomes from the 1000 Genomes Project. These were analyzed using each tool and the ability of each to rank the causative variant as the top hit, in the top 10 or top 50 was recorded. Default settings along with a minor allele frequency cutoff of 1 % were used for all. Analysis was performed using all phenotype annotations (**a**), just three of the terms chosen randomly (**b**), or with two of these three terms made less-specific and two random terms from the whole of the Human Phenotype Ontology (HPO) added (**c**)

Fifty exomes is too small a number to make statistically valid conclusions on the performance of each tool, but we were limited to this number as we wanted to include Phevor and this was only available through manual, web use. However, the results from 1000 exomes run through the other tools (Fig. 2) did not differ much from that seen from 50 exomes, so the results are likely to be representative. In addition, the results are in rough agreement with previously published reports of performance using a similar strategy: 97 % as the top hit using PhenIX or Exomiser, 88 % as the top hit with Phen-Gen, and 95 % in the top 10 for Annovar plus Phevor.

As expected, the tools that took advantage of phenotype data outperformed prioritization tools that rely on variant analysis alone. For the exomes that were based on the 1000 Genomes Project, Exomiser, PhenIX, Phen-Gen and Phevor clearly outperformed eXtasy, with PhenIX looking like the best option when the phenotype is clearly defined and Exomiser performing the best when missing, generalized and atypical phenotypes are present. The same pattern was seen for the analyses of the samples based on our in-house-generated exomes, except that the performance of Phen-Gen decreased dramatically such that it was the worst performing tool. Phen-Gen was unable to prioritize any of the disease variants as the top hit in these samples. Phen-Gen uses a measure of genic intolerance that is based on 1000 Genomes Project data, and it could be that this plays a large part in the impressive performance of this tool when analyzing the simulated 1000 Genomes-based exomes. As shown in Table 2, the dramatic filtering Phen-Gen achieved when working with the 1000 Genomes Project-based exomes was not reproduced for our in-house exomes. This is likely to be primarily related to the fact that frequency data are available for all variants in the 1000 Genomes Project exomes, but in-house data are likely to have 5–10 % 'private' variants with no available frequency data.

In terms of ease of use for the benchmarking, the tools that were available for download and command-line usage were clearly more convenient and suitable for high-throughput analysis. Exomiser, PhenIX and Annovar took 1–2 minutes to run each sample, but Phen-Gen took around 20 minutes and EXtasy took up to 50 minutes. When running on the Annovar pre-filtered results, Phevor takes less than a minute but a lot of initial manual work must be performed to generate the ANNOVAR file, upload it, enter all the HPO terms and launch the analysis.

## Outlook: the future of phenotypic-driven analysis of genomic data

In this review, we have examined contemporary phenotype-driven exome analysis software. We performed an evaluation of several contemporary programs. Although the performance of the programs in tests such as ours is likely to depend on the way testing is performed, our results give a general idea of the performance that may be expected from phenotype-driven analysis of exomes in real experiments. We note, however, that not all individuals undergoing exome sequencing to evaluate a suspected rare disease will have a mutation that can be detected by exome sequencing; for instance, some patients with Mendelian disease may have mutations in distal enhancer sequences [53]. Every simulated patient in our analysis had a mutation that was detectable by exome sequencing, and so the rate of identification of causal mutations by phenotype-driven analysis of real exome data may be lower than that in our simulations. In addition, all of the tools we examined, with the exception of Phen-Gen, are likely to be systematically biased by training on known disease variants, which are almost always in coding regions. Finally, we suggest that the performance of phenotype-driven exome analysis software would be improved by better and more detailed phenotypic annotations [54]. Even with these limitations, however, the performance of programs such as Phevor, eXtasy, Phen-Gen, PhenIX, and Exomiser [5, 38, 41, 49, 50, 55] has clearly demonstrated the value of computational phenotype analysis for the interpretation of exome sequencing data from individuals with rare genetic disease.

While large-scale phenotyping initiatives have become almost routine for model organisms such as the mouse [56], rat [57, 58], and zebrafish [59], similar large-scale efforts for human disease have been lacking. The HPO project [32] and the Monarch Initiative [60] are developing resources towards providing a sound foundation for the annotation and computational analysis of phenotypic abnormalities in human disease and model organisms. A spate of challenges and opportunities remain: for example, improved ontological resources and more detailed annotations are required, especially for conditions such as behavioral abnormalities [33] and for 'new' phenotypes that are observable only with recently introduced technologies, such as abnormalities found upon glycomics analysis or muscle anomalies detectable by magnetic resonance imaging. More detailed phenotyping of larger cohorts of patients together with mutation data may help us to understand genotype–phenotype correlations. In this sense, it is important that the Leiden Open Variation Database (LOVD) software is increasingly capturing phenotype data on individual mutations, and offers the ability to use HPO terms [61].

One of the major goals of computational phenotype analysis of the kind described here is to empower the analysis of NGS data, not only in the context of rare disease but also in the context of personalized medicine. One of the goals of personalized medicine is to classify patients into subpopulations that differ with respect to disease susceptibility, phenotypic or molecular subclass of a disease, or the likelihood of a positive or adverse response to a specific therapy. The related concept of 'precision medicine', whose goal is to provide the best available care for each individual, refers to the stratification of patients into subsets each with a common biological basis of disease, such that stratified medical management is most likely to benefit the patients [62]. All medically relevant disease sub-classifications can be said to have a distinct phenotype, with the understanding that a medical phenotype comprises not only the abnormalities described but also the response of a patient to a certain type of treatment (for example, responsiveness of seizures to valproic acid can be considered to be a phenotype of certain forms of epilepsy). Therefore, comprehensive and precise phenotypic data, combined with ever increasing amounts of genomic data, appear to have an enormous potential to accelerate the identification of clinically actionable complications and of disease subtypes with prognostic or therapeutic implications.

The algorithms presented in this review probably represent only the first generation of increasingly powerful computational tools that will combine phenotype analysis and the investigation of genetic variants identified by WES or whole genome sequencing with the study of human disease and the practice of medicine.

## Box 1. Prominent exome sequencing projects in the field of rare disease research

A number of large-scale, multicenter projects have emerged in recent years that aim to use whole exome sequencing (WES) to discover novel disease-associated genes and to improve the diagnosis and treatment of rare hereditary diseases. These include:

- **Care4Rare** (http://care4rare.ca/). This project has emerged from the Canadian FORGE (Finding of Rare Disease Genes) initiative, which has been able to identify disease-causing variants for 146 of the 264 disorders studied over a 2-year period, with up to 67 novel disease-associated genes being characterized [63].
- **Centers for Mendelian Genomics (CMG)** (http://www.mendelian.org/). A group of sequencing centers funded by the National Institutes of Health has established three CMGs (Baylor-Johns Hopkins CMG, the University of Washington CMG and the Yale CMG) [64].
- **Undiagnosed Disease Program of the National Institutes of Health** (http://www.genome.gov/27550959). The Undiagnosed Disease Program was founded with the goal of achieving a diagnosis for patients who remained undiagnosed after an exhaustive workup and to discover new disorders that would provide insight into mechanisms of disease [65].
- **The UK 100,000 Genomes Project** (http://www.genomicsengland.co.uk/). This project includes a major focus on rare inherited diseases with the goal of introducing genomics diagnostics into the mainstream healthcare system for the benefit of patients and researchers.
- **DECIPHER (DatabasE of genomiC varIation and Phenotype in Humans using Ensembl Resources)** (https://decipher.sanger.ac.uk/). This resource has been in operation since 2004 and represents a community driven database of array comparative genomic hybridization (CGH) and WES data that can be used for genomic matchmaking [66].
- **The Deciphering Developmental Disorders (DDD) study** (http://www.ddduk.org/) has the goal of improving diagnostics of developmental disorders in children by means of array CGH and next-generation sequencing methods. The program has achieved a diagnostic yield of 27 % among 1133 previously investigated yet undiagnosed children who have developmental disorders [67].
- **The Global Alliance for Genomics and Health** coordinates several groups that are involved in genomic matchmaking, which allows physicians to search for patients with similar genotypes and phenotypes to facilitate and accelerate novel disease-associated gene discovery. Many of these databases, such as PhenomeCentral (https://phenomecentral.org/), use phenotype analysis.

The analysis of data in these and other projects benefits greatly from other collections of exome data that allow the frequency of variants in the population to be estimated (for instance, in order to filter out variants whose population frequency exceeds a certain threshold). These include the NHLBI-ESP 6500 exome project (https://esp.gs.washington.edu/drupal/), the Exome Aggregation Consortium (ExAC) (http://exac.broadinstitute.org/), and the 1000 Genomes Project [23].

## Box 2. Selection of tools used for the analysis of variants found in whole exome sequencing data

**Variant annotation tools** translate the genomic coordinates of variants given by variant call format (VCF) files (which are commonly used in exome sequencing) into the corresponding transcript-based annotations. ANNOVAR annotates variants in this way and performs tasks such as examining their functional consequence on genes. In addition, this tool performs functional annotation of the variants with respect to a number of attributes [22]. Jannovar performs such annotation as well as pedigree-based analysis and can also be used as a Java programming library [21].

**Pathogenicity prediction programs** use computational analysis to assess the potential impact of amino acid substitutions, and in some cases other categories of variants, on protein function. Sorting Intolerant from Tolerant (SIFT) uses sequence homology to predict the likelihood that an amino acid substitution will have an adverse effect on protein function [68]. Polymorphism Phenotyping v2 (PolyPhen-2) predicts the impact of amino acid substitutions on the stability and function of affected proteins using structural and comparative evolutionary comparisons [25]. MutationTaster uses Bayesian methodologies to predict the relevance of a wide range of variants [69]. The Combined Annotation scoRing toOL (CAROL) combines the predictions of PolyPhen-2 and SIFT [70]. The Combined Annotation-Dependent Depletion (CADD) integrates a large number of sequence and genomic attributes to train a support vector machine to predict deleteriousness [27]. Genomic Evolutionary Rate Profiling (GERP) is a method to assess regions that have been subject to purifying selection and are enriched for functional elements [26].

Variant annotation pathogenicity prediction tools are used to assess the potential relevance of variants in WES data. In phenotype-driven exome analysis, the final ranking of the genes that contain these variants is performed using phenotypic analysis according to the algorithms described for the several programs.

## Additional file

Additional file 1: Table S1. Detailing the diseases and variants used for the benchmarking of the 50 exomes in Fig. 1.

## Abbreviations

CADD: Combined Annotation-Dependent Depletion
CAROL: Combined Annotation scoRing toOL
CGH: comparative genomic hybridization
DO: Disease Ontology
CMG: Center for Mendelian Genomics
ESP: NHLBI-ESP 6500 exome project
GERP: Genomic Evolutionary Rate Profiling
GO: Gene Ontology
HGMD: Human Genome Mutation Database
HPO: Human Phenotype Ontology
MAF: minor allele frequency
MPO: Mammalian Phenotype Ontology
NGS: next-generation sequencing
Phevor: Phenotype Driven Variant Ontological Re-ranking tool
PolyPhen: Polymorphism Phenotyping
SIFT: Sorting Intolerant from Tolerant
VAAST: Variant Annotation, Analysis, Search Tool
VCF: variant call format
WES: whole exome sequencing
