# Supplementary material for: Phenotype-driven strategies for exome prioritization of human Mendelian disease genes
Source: Genome Med. 2015 Jul 30;7(1):81. doi: 10.1186/s13073-015-0199-2 (PMC4520011; doi:10.1186/s13073-015-0199-2)
Supplement: Additional file 1: Table S1. — Detailing the diseases and variants used for the benchmarking of the 50 exomes in Fig. 1. [file 13073_2015_199_MOESM1_ESM.docx]

**Additional Table 1:** Diseases and variants used for the benchmarking of the 50 exomes in Fig. 1

| **OMIM ID** | **OMIM term** | **Gene** | **Inheritance** | **Chromosome** | **Position** | **Ref** | **Alt** |
| --- | --- | --- | --- | --- | --- | --- | --- |
| OMIM:102700 | Severe Combined Immunodeficiency, Autosomal Recessive, T Cell-Negative, B Cell-Negative, Nk Cell-Negative, Due To Adenosine Deaminase Deficiency | ADA | AR | 20 | 43249032 | G | A |
| OMIM:113800 | Epidermolytic Hyperkeratosis | KRT10 | AD | 17 | 38975784 | A | G |
| OMIM:118220 | Charcot-Marie-Tooth Disease, Demyelinating, Type 1a | PMP22 | AD | 17 | 15134277 | A | C |
| OMIM:120435 | Lynch Syndrome I | MSH2 | AD | 2 | 47630331 | A | C |
| OMIM:131100 | Multiple Endocrine Neoplasia, Type I | MEN1 | AD | 11 | 64571903 | A | G |
| OMIM:133020 | Erythermalgia, Primary | SCN9A | AD | 2 | 167083097 | A | C |
| OMIM:159900 | Myoclonic Dystonia | SGCE | AD | 7 | 94230139 | G | A |
| OMIM:168000 | Paragangliomas 1 | SDHD | AD | 11 | 111957632 | A | G |
| OMIM:170500 | Hyperkalemic Periodic Paralysis | SCN4A | AD | 17 | 62019291 | G | A |
| OMIM:201100 | Acrodermatitis Enteropathica, Zinc-Deficiency Type | SLC39A4 | AR | 8 | 145638174 | C | A |
| OMIM:202110 | Adrenal Hyperplasia, Congenital, Due To 17-Alpha-Hydroxylase Deficiency | CYP17A1 | AR | 10 | 104590499 | C | T |
| OMIM:210720 | Microcephalic Osteodysplastic Primordial Dwarfism, Type Ii | PCNT | AR | 21 | 47766060 | G | T |
| OMIM:210900 | Bloom Syndrome | BLM | AR | 15 | 91292809 | C | A |
| OMIM:212050 | Candidiasis, Familial, 2 | CARD9 | AR | 9 | 139264814 | G | A |
| OMIM:214450 | Griscelli Syndrome, Type 1 | MYO5A | AR | 15 | 52643564 | G | A |
| OMIM:214450 | Griscelli Syndrome, Type 1 | MYO5A | AR | 15 | 52643564 | G | A |
| OMIM:219000 | Fraser Syndrome | FREM2 | AR | 13 | 39358840 | G | A |
| OMIM:223000 | Lactase Deficiency, Congenital | LCT | AR | 2 | 136558209 | C | A |
| OMIM:226730 | Epidermolysis Bullosa Junctionalis With Pyloric Atresia | ITGB4 | AR | 17 | 73723307 | T | C |
| OMIM:227300 | Factor V And Factor Viii, Combined Deficiency Of, 1 | LMAN1 | AR | 18 | 56998723 | A | G |
| OMIM:231070 | Geroderma Osteodysplasticum | GORAB | AR | 1 | 170508425 | G | T |
| OMIM:232500 | Glycogen Storage Disease Iv | GBE1 | AR | 3 | 81584397 | T | C |
| OMIM:233700 | Granulomatous Disease, Chronic, Autosomal Recessive, Cytochrome B-Positive, Type I | NCF1 | AR | 7 | 74191665 | G | A |
| OMIM:235200 | Hemochromatosis, Type 1 | HFE | AR | 6 | 26087686 | G | C |
| OMIM:235510 | Hennekam Lymphangiectasia-Lymphedema Syndrome | CCBE1 | AR | 18 | 57134004 | A | G |
| OMIM:236250 | Homocystinuria Due To Deficiency Of N(5,10)-Methylenetetrahydrofolate Reductase Activity | MTHFR | AR | 1 | 11850952 | C | T |
| OMIM:240600 | Glycogen Storage Disease 0, Liver | GYS2 | AR | 12 | 21693408 | C | T |
| OMIM:241850 | Hypothyroidism, Athyroidal, With Spiky Hair And Cleft Palate | FOXE1 | AR | 9 | 100616342 | T | C |
| OMIM:246650 | Lipase Deficiency, Combined | LMF1 | AR | 16 | 904642 | C | T |
| OMIM:253000 | Mucopolysaccharidosis, Type Iva | GALNS | AR | 16 | 88880849 | A | C |
| OMIM:259900 | Hyperoxaluria, Primary, Type I | AGXT | AR | 2 | 241808284 | T | C |
| OMIM:262850 | Alpha-2-Plasmin Inhibitor Deficiency | SERPINF2 | AR | 17 | 1657583 | G | A |
| OMIM:267500 | Reticular Dysgenesis | AK2 | AR | 1 | 33478946 | G | A |
| OMIM:274600 | Pendred Syndrome | SLC26A4 | AR | 7 | 107302089 | G | C |
| OMIM:274800 | Thyroid Dyshormonogenesis 4 | IYD | AR | 6 | 150710610 | C | T |
| OMIM:274900 | Thyroid Dyshormonogenesis 5 | DUOXA2 | AR | 15 | 45409299 | T | C |
| OMIM:600081 | Vitamin D Hydroxylation-Deficient Rickets, Type 1b | CYP2R1 | AR | 11 | 14907393 | A | G |
| OMIM:601626 | Leukemia, Acute Myeloid | SH3GL1 | AD | 19 | 4364141 | G | A |
| OMIM:602083 | Usher Syndrome, Type If | PCDH15 | AR | 10 | 55581929 | T | G |
| OMIM:602088 | Nephronophthisis 2 | INVS | AR | 9 | 102992057 | G | A |
| OMIM:604290 | Aceruloplasminemia | CP | AR | 3 | 148894111 | C | T |
| OMIM:604403 | Generalized Epilepsy With Febrile Seizures Plus, Type 2 | SCN1A | AD | 2 | 166847915 | T | C |
| OMIM:605259 | Spinocerebellar Ataxia 13 | KCNC3 | AD | 19 | 50826942 | C | T |
| OMIM:607271 | Caspase 8 Deficiency | CASP8 | AR | 2 | 202141631 | C | T |
| OMIM:607625 | Niemann-Pick Disease, Type C2 | NPC2 | AR | 14 | 74947410 | G | A |
| OMIM:608810 | Myopathy, Myofibrillar, 2 | CRYAB | AD | 11 | 111779505 | C | T |
| OMIM:610153 | Deafness, Autosomal Recessive 49 | MARVELD2 | AR | 5 | 68728915 | C | T |
| OMIM:610475 | Pigmented Nodular Adrenocortical Disease, Primary, 2 | PDE11A | AD | 2 | 178534265 | C | T |
| OMIM:610915 | Osteogenesis Imperfecta, Type Viii | LEPRE1 | AR | 1 | 43232251 | G | T |
| OMIM:611038 | Microphthalmia, Isolated 3 | RAX | AR | 18 | 56936702 | C | T |
